# Effect of Facilitation of Local Stakeholder Groups on Equity in Neonatal Survival; Results from the NeoKIP Trial in Northern Vietnam

**DOI:** 10.1371/journal.pone.0145510

**Published:** 2015-12-29

**Authors:** Mats Målqvist, Dinh Phuong Thi Hoa, Lars-Åke Persson, Katarina Ekholm Selling

**Affiliations:** 1 International Maternal and Child Health, Department of Women’s and Children’s Health, Uppsala University, Uppsala, Sweden; 2 Hanoi School of Public Health, Hanoi, Vietnam; Institute for Health & the Environment, UNITED STATES

## Abstract

**Background:**

To operationalize the post-MDG agenda, there is a need to evaluate the effects of health interventions on equity. The aim of this study is to evaluate the effect on equity in neonatal survival of the NeoKIP trial (ISRCTN44599712), a population-based, cluster-randomized intervention trial with facilitated local stakeholder groups for improved neonatal survival in Quang Ninh province in northern Vietnam.

**Methods:**

Semi-structured interviews were conducted with all mothers experiencing neonatal mortality and a random sample of 6% of all mothers with a live birth in the study area during the study period (July 2008-June 2011). Multilevel regression analyses were performed, stratifying mothers according to household wealth, maternal education and mother’s ethnicity in order to assess impact on equity in neonatal survival.

**Findings:**

In the last year of study the risk of neonatal death was reduced by 69% among poor mothers in the intervention area as compared to poor mothers in the control area (OR 0.31, 95% CI 0.15–0.66). This pattern was not evident among mothers from non-poor households. Mothers with higher education had a 50% lower risk of neonatal mortality if living in the intervention area during the same time period (OR 0.50, 95% CI 0.28–0.90), whereas no significant effect was detected among mothers with low education.

**Interpretation:**

The NeoKIP intervention promoted equity in neonatal survival based on wealth but increased inequity based on maternal education.

## Introduction

**Fig 1:**
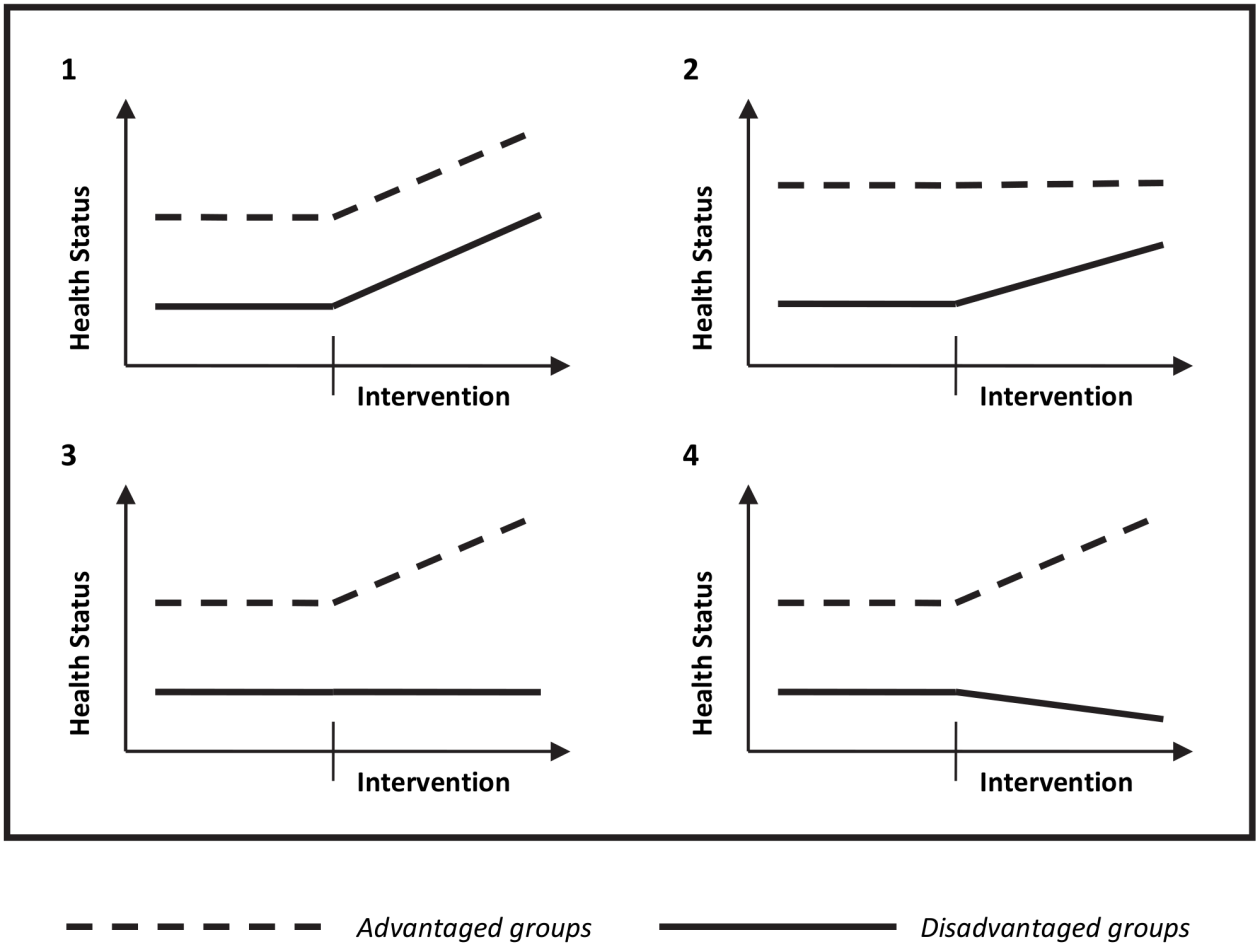
Possible impacts on equity by public health interventions.

Achieving health equity is a major challenge for policy makers and health care planners around the globe [1] as well as a major challenge for the coming post-MDG agenda [2,3]. No one disputes that inequalities in health outcomes exist, but more focus on how to overcome the socially unjust disparities, i.e. inequities, is needed [4]. Neonatal survival has been shown to be highly inequitable [5,6], even if recent analyses have shown promising trends with reduced inequity in neonatal survival [7]. The neonatal period as such has long been neglected. However, neonatal survival in a global context has just recently started to improve after a long period of stagnant development [8,9]. Simple and cost-effective interventions like exclusive breast-feeding, resuscitation at birth and knowledge about infection danger signs have started to be implemented through international and national initiatives [8]. Even if there is still a long way to go before the large amount of preventable deaths in the neonatal period has been eliminated, the field of implementation science is growing, evaluating methods to translate evidence-based knowledge into practice [10]. However, few intervention studies have evaluated the impact of selected interventions or packages of interventions on equity [11–13]. Theoretically an effective intervention of any kind might have four different impacts on equity (Fig 1) [14]: (1) to maintain the inequity gap by an equal impact of the intervention on all groups in society, or (2) to reduce inequity by a larger impact of the intervention on disadvantaged groups, or (3) to increase inequity by a larger impact of the intervention on the already better-off, or, finally, (4) to increase inequity by exacerbating the situation for disadvantaged groups.

Inequity is commonly defined and measured at a structural level. The Commission on Social Determinants of Health (CSDH) has developed a framework to describe the components and pathways of inequitable health outcomes [15]. The central feature of this framework is that social position is the origin and driver of health inequities. The social position is in turn expressed by and a result of structural factors such as gender, ethnicity, education and income. Therefore when measuring inequity in health, these variables are used as independent variables due to their close relationship to social position. The structural determinants’ effect on health is mediated through a set of intermediary determinants grouped by CSDH into four different categories: material circumstances, behavioral factors, psychosocial factors and health system factors. It is worth pointing out that the intermediary determinants are only in the causal pathway between social position and health inequity and cannot by themselves generate inequity. For example, to live in a slum area is not in itself a cause of inequity but a result of the inhabitants’ social position as expressed by income, education and ethnicity.

### The NeoKIP trial

We conducted an intervention for improved neonatal survival in Quang Ninh province in northern Vietnam[16]. Through the facilitation of monthly local stakeholder groups focusing on the perinatal health situation in the respective commune the risk of neonatal death was decreased by around 40% in intervention communes after three years of intervention[17]. The field trial, called NeoKIP (Neonatal Health—Knowledge Into Practice, ISRCTN44599712) was conducted by facilitators trained by the research group to stimulate problem-solving using the PDSA (Plan-Study-Do-Act) cycle in groups consisting of local stakeholders, such as commune health center staff, representatives from the Women’s Union and local decision makers [16]. The trained facilitators were local women recruited from the Women’s Union without prior health care training. These local stakeholder groups identified and prioritised local problems in relation to maternal and newborn care and performed targeted activities between meetings. There was no specific equity focus in the intervention design, such as targeting of disadvantaged groups. However, since all groups were encouraged to define and target challenges of neonatal health in their respective communes, there was an inherent equity dynamic in the intervention, since it can be assumed that the disadvantaged groups might be targeted for improvement efforts. Group members were encouraged to seek information on evidence-based practices from available resources such as the National Guidelines on Reproductive Health launched by the Ministry of Health in 2002 and revised in 2009. No specific training on maternal and newborn care was provided by the NeoKIP project, but the MoH continuously worked to improve maternal and newborn health in the whole study area. The 90 communes in the study area were randomized using a one-stage cluster sampling with probability proportional to size (PPS) of the clusters. Intervention activities were carried out in 44 communes in eight districts in Quang Ninh province and 46 communes in the same districts were used as controls. Baseline characteristics of intervention and control communes were similar, even if a higher share of poor families and mothers lacking formal education could be observed in control communes. Control communes received no additional information or training apart from what was provided by the Ministry of Health through the Provincial Health Bureau of Quang Ninh province. A main outcome paper and process evaluation of the intervention has been published elsewhere [17,18].

### Objective

The objective of this study was to ascertain the impact of the NeoKIP trial on equity in neonatal survival. The intervention was shown to be effective, reducing the neonatal mortality rate in intervention communes in the third year of intervention [17]. Further analysis to investigate if this intervention was equitable and how neonatal survival chances were affected for the disadvantaged groups of poor, ethnic minorities and mothers with low education is needed.

## Methods

### Setting

Quang Ninh province is situated in northern Vietnam, bordering China to the north and stretching along the vast coastline of the South China Sea. More than one million inhabitants, predominantly from the ethnic majority Kinh group, live mostly clustered around the main road, which is a major trading route to China. Ethnic minorities in this province are found, as in the rest of Vietnam, in higher density in the more remote and mountainous areas. The rapid economic development of Vietnam is reflected in large investments in mining and tourism, the main economic industries of Quang Ninh. A large coal and chalk mining industry attracts large numbers of migrant workers and the Ha Long Bay area, a UNESCO World Heritage site, is a popular destination for a growing number of tourists.

The health care system follows the administrative borders of Quang Ninh province with at least one commune health centre in each of the province’s 184 communes and one district hospital in 13 out of the 14 districts of the province. In Ha Long City, the province capital, there is a tertiary hospital, the Province Hospital, and the Vietnam Sweden General Hospital (UBGH), a regional transfer hospital directly under the administrative authority of the Ministry of Health, is located in Uong Bi City. Both the Province Hospital and UBGH have well-functioning neonatology departments, but there is also a possibility to transfer patients to the National Paediatrics Hospital in Hanoi if necessary.

### Sample

**Fig 2:**
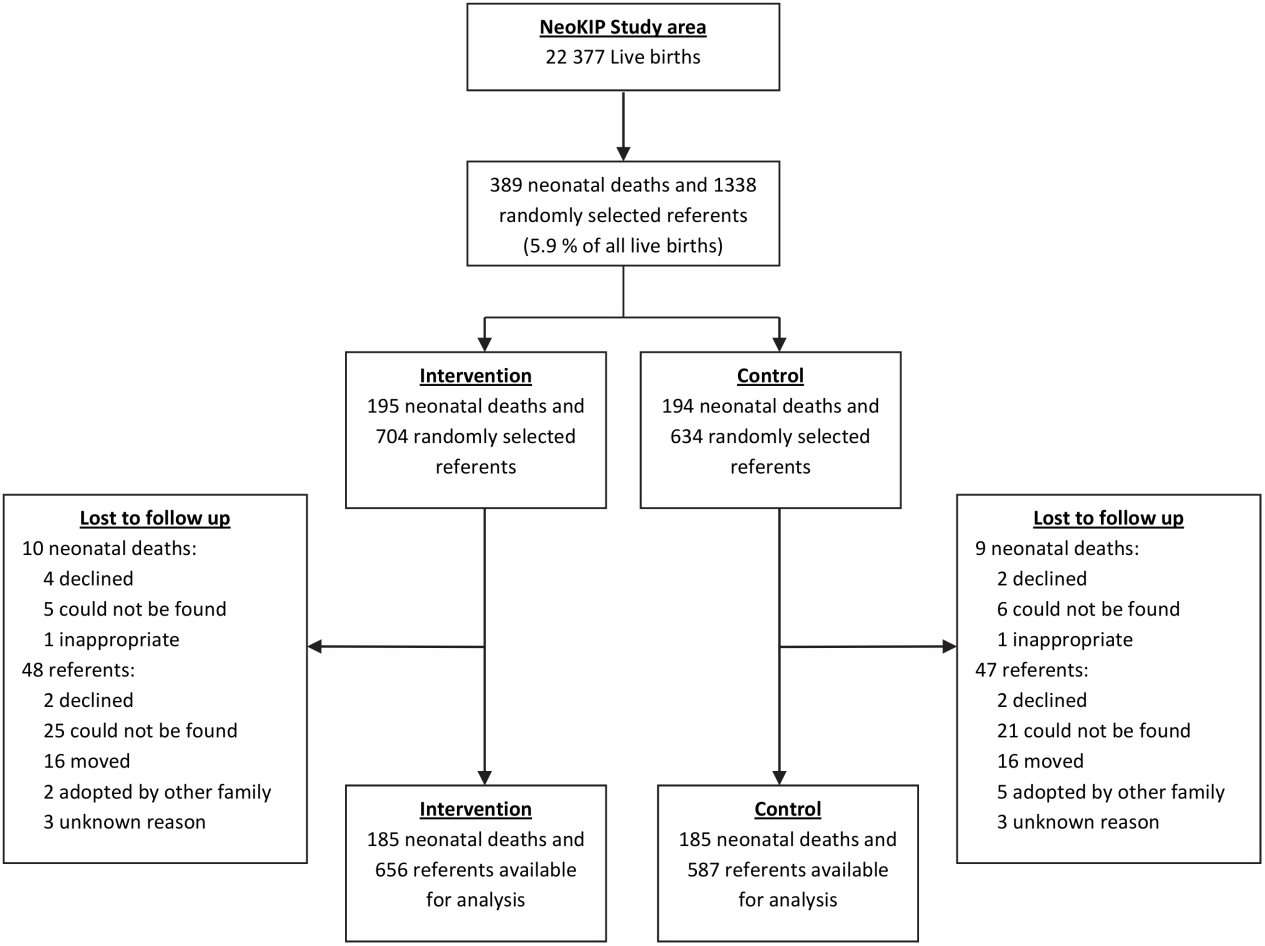
Flow chart of referents and case interviews.

During the study period, July 2008 until June 2011, all live births and neonatal deaths in the study area were recorded, resulting in 22,377 live births and 389 neonatal deaths. All mothers who had lost a newborn during the neonatal period were interviewed. In addition, mothers of a representative sample of 6% of all the live births were randomly selected as referents and interviewed. After loss to follow-up, 370 neonatal death case mothers and 1,243 referent mothers were interviewed and eligible for data analysis (Fig 2) (S1 Dataset). Trained data collectors using a semi-structured interview form performed interviews six to eight weeks after delivery.

### Ethical considerations

Informed consent was obtained from all mothers and recorded on interview forms. A verbal procedure for informed consent was considered more appropriate in the Vietnamese setting, making it less official so as not to jeopardize the interviewees’ trust. This procedure as well as the full study was approved by the Ministry of Health in Vietnam, the Provincial Health Bureau in Quang Ninh and the Research Ethics Committee at Uppsala University, Sweden.

### Data analysis

Multi-level logistic regression (or generalized linear mixed models GLMM) quantified the associations between the structural determinants, i.e. ethnicity (Kinh/other), maternal education (>5 years/< = 5 years), and household economic status (poor/non-poor), and the outcome neonatal mortality. Household economic status was estimated through the use of an asset and household status index using principal component analysis to calculate a wealth score based on referent mothers. Households with scores in the lowest quintile were considered poor and the rest non-poor. The results are presented by means of odds ratios (OR) and 95% confidence intervals (CI). The R package ‘lme4’ [19] was used in all calculations. In Table 1, the three structural determinants together with intervention (yes/no) were simultaneously included in the same model. We also tested for interaction between the intervention variable and ethnicity in this model. In Table 2, the association between the intervention variable and neonatal mortality was explored for the subset of the third intervention year only, and by stratification for each of the structural determinants. In Table 3, each structural determinant was simultaneously included in one model, and stratifications were made for intervention year (1 or 3), and intervention group (intervention/control). Throughout the analysis, the structural determinants as well as the intervention variable were treated as fixed factors, nested within the random factor commune (i.e. the clusters).

**Table 1:** Multi-level logistic regression model with maternal ethnicity, household economic status and maternal education level with adjusted odds ratios for neonatal mortality in NeoKIP study area in Quang Ninh province,Vietnam. Adjusted for cluster randomization by means of Generalized linear mixed models (GLMM) with all variables in table as well as intervention.

	Ref (n)	Cases (n)	OR^	95% CI
Kinh	788	128	Ref	
Minority	455	242	1.88^†^	1.38–2.72
Non-Poor	994	186	Ref	
Poor	249	184	2.56^†^	1.88–3.50
Primary school or higher	804	170	Ref	
No primary school	439	200	1.54*	1.11–2.13

^(yes/no) included simultaneously as fixed factors in the model, nested under the random factor cluster.

^†^p < 0.001,

*p = 0.01

**Table 2:** Odds ratios (OR) for neonatal mortality in third year of NeoKIP intervention stratified by structural determinants in Quang Ninh province,Vietnam. Adjusted for cluster randomization by means of Generalized linear mixed models (GLMM).

	Ref (n)	Cases (n)	OR^	95% CI
**Ethnicity**				
*** Kinh***				
Control	115	21	Ref	
Intervention	161	15	0.47	(0.19–1.18)
*** Minority***				
Control	73	50	Ref	
Intervention	76	29	0.63	(0.33–1.23)
**Household economic status**				
*** Non-poor***				
Control	158	27	Ref	
Intervention	200	27	0.76	(0.39–1.48)
*** Poor***				
Control	30	44	Ref	
Intervention	37	17	0.31^†^	(0.15–0.66)
* * ***Primary school or higher***				
Control	131	36	Ref	
Intervention	188	21	0.50*	(0.28–0.90)
* * ***No primary school***				
Control	57	35	Ref	
Intervention	49	23	0.73	(0.23–2.30)

^(yes/no) included simultaneously as fixed factors in the model, nested under the random factor cluster.

* p < 0.05,

^†^p < 0.001

**Table 3:** Multi-level logistic regression models with structural determinantsdisplaying adjusted odds ratios (OR, 95% Confidence intervals within brackets) for neonatal mortality in Quang Ninh province,Vietnam, stratified by NeoKIP intervention and control area. Adjusted for cluster randomization by means of Generalized linear mixed models (GLMM) with all variables in table included simultaneously as fixed factors in the models in the different strata, nested under the random factor cluster.

	Intervention		Control	
	OR at baseline (first year)	OR in third year of intervention	OR at baseline (first year)	OR in third year of intervention
Kinh	Ref	Ref	Ref	Ref
Minority	2.38* (1.20–4.71)	3.42* (1.19–9.89)	1.72 (0.82–3.60)	1.44 (0.70–2.97)
Non-Poor	Ref	Ref	Ref	Ref
Poor	2.31* (1.12–4.78)	1.60 (0.60–4.26)	1.52 (0.76–3.07)	6.28^†^ (3.17–12.4)
Primary school or higher	Ref	Ref	Ref	Ref
No primary school	0.83 (0.37–1.85)	2.60 (0.84–8.05)	2.26* (1.11–4.61)	2.31* (1.10–4.85)

*p < 0.05,

^†^p < 0.001

## Results

In total, 185 mothers to neonatal mortality death cases were interviewed in both intervention and control areas. Six hundered and fifty-six (656) referent mothers interviewed lived in intervention area and 587 of the interviewed referent mothers lived in the control area (Fig 2). Over the entire study period no significant effect of the intervention could be seen (OR 1.09, 95% CI 0.81–1.24), adjusted for household economic status, maternal ethnicity and education. Neither was any significant effect seen in the first and second year of intervention. However, in the third year of intervention there was a 44% risk reduction of neonatal death experienced by mothers living in the intervention area compared to mothers living in the control area (OR 0.56, 95% CI 0.36–0.89, adjusted for household economic status, maternal ethnicity and education).

Household economic status, maternal ethnicity and education were all associated with neonatal mortality (Table 1), indicating inequity in neonatal survival based on these structural determinants. When stratifying the structural determinants in the third year of intervention there was a positive effect of the intervention among mothers from poor households and mothers who had completed primary school or higher (Table 2). Thus the intervention effect among mothers from poor households showed an 69% risk reduction of neonatal mortality compared to poor mothers living in the control area (OR 0.31, 95% CI 0.15–0.66). No significant effect of the intervention was seen among mothers from non-poor households. However, the opposite was detected when stratifying for education, where mothers with higher education benefited from the intervention (OR 0.50, 95% CI 0.28–0.90), whereas no effect was detected among mothers with low education. When stratifying for ethnicity no significant reduction of neonatal mortality could be detected.

In order to estimate the magnitude of the impact on equity by the intervention, the equity gap in neonatal survival described in Table 1 was divided over the three years of intervention. There was increasing inequity in neonatal survival based on household economic status in the control area, whereas there was an opposite trend in the intervention area (Table 3). When comparing inequity in neonatal survival based on household economic status in the third year of intervention there was a discrepancy in inequity between the intervention (OR 1.60, 95% CI 0.60–4.26) and control (OR 6.28, 95% CI 3.17–12.4) areas. Regarding the effects on equity based on maternal education, the opposite has previously been demonstrated. This is illustrated in Table 3, where the trend is that higher education becomes protective in the intervention area, expressed as an increased risk of neonatal mortality for mothers with low education (OR 2.30, 95% CI 1.10–4.85).

## Discussion

We have estimated the impact of a community-based intervention for improved neonatal survival on equity. The NeoKIP intervention has been shown effective and has demonstrated remarkable results in the third year of intervention [17]. By this extended analysis focusing on structural determinants and their association to the outcome, we have come up with contradictory results in relation to equity. Poor mothers benefited from the intervention, while no significant results could be shown for the non-poor. In relation to income the intervention is thus promoting equity. This is in line with a previous study evaluating effects of similar participatory interventions in India [12] and Nepal [13]. However, we have also demonstrated that mothers with higher education benefited from the intervention whereas mothers with low education did not, with an increasing inequity based on education as a result. The community-based approach of the intervention, where local stakeholder groups were asked to define the problems in maternal and newborn health in their respective local context, might be one of the explanations why the largest improvement was seen among the economically disadvantaged. This group is already perceived as disadvantaged and in need of targeting. Therefore it is reasonable to assume that the local stakeholder groups would focus their efforts on them. The NeoKIP intervention is also, as stated by the acronym, about translating knowledge into practice. This might be an explanation for the result that mothers with higher education benefited from the intervention whereas a similar effect was not found among mothers with lower education. Even if the local stakeholder groups chose to target the poor and minorities, the choice of actions and measures taken were more oriented towards increasing knowledge levels and awareness among pregnant women than about changing practice. It has been shown before that knowledge-based interventions primarily reach those who are better educated [20,21].

The case-referent design used for this study is viable and appropriate, and the effects of the intervention based on the representative sample of all live births is in line with the effect estimate performed on the population-based material. By using this design we have been able to retrieve more information from mothers than would have been feasible to acquire in a population-based sample, such as education level and income. Household economic status was assessed by the use of an asset index and calculated by principal component analysis. It can be argued that this method has some drawbacks, but in general the method is sound [22]. A mix of assets and household characteristics further strengthens the validity of the method. There is no pre-determined level at which to draw the poverty line when using an asset index. Usually the arbitrary choice is at the 40^th^ percentile [23]. We have, however, chosen to draw the poverty line at the 20^th^ percentile since economic growth in Vietnam over the past decade has benefitted the poorest the least [24]. This development has resulted in a more prominent bottom inequity, in which a small minority is much worse off than everyone else. Drawing the poverty line at the 20^th^ percentile leaves a large enough number of subjects to be able to do an appropriate analysis while at the same time not diluting the most disadvantaged group. The cut off between high and low maternal education levels is arbitrary and considering that the school system has good coverage in the study area we decided to use primary school completion as the cut-off point. Similar results as the ones presented here were reached when setting the cut off at not completing primary school versus completing primary school or above. Concerning the ethnicity variable it might be argued that the different ethnic minority groups are heterogeneous and that grouping them together as “minority” will not give the different cultural traits justice [25]. Since this study wanted to analyse equity, though, it is not primarily the cultural expressions of the ethnic minorities that are interesting but rather the social position derived from minority status. As such, all minorities fall in the same category of being disadvantaged.

In our analysis we have departed from the CSDH framework on determinants of inequity in health. The notion that social position is the main basis for inequity puts a special emphasis on the structural determinants of health and income is often used as the most important determinant in equity analyses. We have included ethnicity and education as well, since maternal education is well known to be influential for maternal and child health outcomes [20], and ethnicity has been shown to be highly important in the Vietnamese setting [5]. We have not included any of the intermediary determinants in our analysis. This was a deliberate choice since the main objective was to investigate whether or not the intervention at hand was equitable, and not to explain the working mechanisms of its positive effects on neonatal survival.

Despite the simplicity of the required methodsand the importance of achieving equity in health, there are very fewintervention trials evaluating effects on equity. When reporting effects of different intervention studies it is almost always performed at an aggregate level. One reason is of course that the sample size is many times not sufficient to do the required stratification. Even in this study we would have benefited from a larger sample in order to reach significant results when comparing odds ratios between baseline and final year in the intervention area (Table 3). In order to promote equity analyses in intervention trials this perspective must be taken into consideration at the planning stage. The identification of disadvantaged groups and the method of assessing structural determinants as well as sample size are necessary issues to consider prior to the start of an intervention.

## Conclusion

We have performed an equity analysis of an intervention for improved neonatal survival. With simple and straightforward statistical methods we have managed to show how this intervention impacted disadvantaged groups. The results suggest that the NeoKIP intervention could serve as a possible model for scale-up in order to address inequity in relation to economic status. However, further adaptation of the intervention model to address uneducated mothers is also needed.

## Ethical Approval

The study was approved by the Ministry of Health in Vietnam, the Provincial Health Bureau in Quang Ninh and the Research Ethics Committee at Uppsala University, Sweden.

## Supporting Information

S1 Dataset SPSS file containing NeoKIP data for analysis. (SAV)
